# Joint line rim-plate combined with horizontal belt plate in the treatment of hyperextension tibial plateau fracture: our clinical and radiological results

**DOI:** 10.3389/fsurg.2025.1725660

**Published:** 2025-11-18

**Authors:** Yanqing Gu, Cheng Ma, Lei Zhao, Chunzhi Jiang

**Affiliations:** 1 Department of Orthopedics, Nanjing First Hospital, Nanjing Medical University, Nanjing, Jiangsu, China; 2 Department of Orthopedics, The People’s Hospital of Shimen County (Shimen Hospital of Changsha Medical University), Shimen, Hunan, China

**Keywords:** hyperextension tibial plateau fracture, joint line rim-plate, medial-lateral approach, medial-lateral approaches, locking plates

## Abstract

**Objective:**

To evaluate the efficacy of combined medial and lateral approaches with horizontal belt plate fixation for treating hyperextension-type bicondylar tibial plateau fractures.

**Methods:**

A retrospective analysis was conducted on 10 patients with hyperextension-type bicondylar tibial plateau fractures treated between March 2023 and March 2024 using a combined medial-lateral approach and anterior joint line rim-plate fixation. During surgery, the infrapatellar tendon was released to create a horizontal rim-plate channel. A pre-contoured tubular locking plate was fixed anteriorly to the tibial plateau. A T-shaped main plate was placed on the medial or lateral side based on fracture patterns. Screws from medial, lateral, and anterior plates formed a “fence-like” structure.

**Results:**

All 10 patients achieved bony union with an average follow-up of 14 months. Postoperative radiographs demonstrated restored posterior tibial slope angles. The average Hospital for Special Surgery (HSS) knee score was 88.7, with knee range of motion averaging 113°. No wound complications or implant failures were observed.

**Conclusion:**

Preliminary results indicate that combined medial-lateral approaches with horizontal belt plate fixation are a safe and effective option for hyperextension-type bicondylar tibial plateau fractures. Restoration of lower limb alignment, correction of posterior tibial slope, and joint surface congruity are critical for optimal outcomes.

## Introduction

Tibial plateau fractures, commonly caused by high-energy trauma or low-energy falls in osteoporotic patients, involve articular surfaces through axial loading, lateral stress, or combined mechanisms (1). Hyperextension-type bicondylar tibial plateau fractures are rare, accounting for approximately 3% of all tibial plateau fractures (2, 3). Firoozabadi et al. (4) first systematically described 23 cases in 2016, highlighting their unique injury mechanisms and clinical challenges. Gonzalez et al. (5) reported poorer prognoses for these fractures, including lower functional scores and higher rates of post-traumatic arthritis. Despite satisfactory union rates, functional recovery remains suboptimal compared to other tibial plateau fractures (4, 6).

Conventional fixation strategies for hyperextension-type fractures rely on anteromedial and/or anterolateral locking plates. However, an anterior “bare zone” between these plates often lacks adequate support, risking secondary displacement. Sun et al. (7) introduced a novel approach using a rim-plate to reinforce this region, achieving promising outcomes. Building on this concept, our technique emphasizes a “fence-like” screw arrangement from medial, lateral, and anterior plates to enhance horizontal support and cortical compression.

This study reviews 10 cases of hyperextension-type bicondylar tibial plateau fractures treated with joint line rim-plate fixation and anterior bone grafting, demonstrating satisfactory clinical and radiological outcomes.

## Methods

### Patient selection and preoperative evaluation

A retrospective analysis included patients diagnosed with hyperextension-type bicondylar tibial plateau fractures and treated with joint line rim-plate fixation between March 2023 and March 2024. Hyperextension-type bicondylar tibial plateau fracture was characterized by the medial and lateral depression of the anterior plateau along with a reduced posterior slope angle. Exclusion criteria included pathological/chronic fractures, polytrauma, or vascular/neurological injuries. Ethical approval was obtained from Nanjing First Hospital. Preoperative imaging (x-ray, CT, MRI) confirmed fracture morphology and excluded ligamentous or vascular injuries. All patients provided written informed consent prior to surgery. The study was approved by the Ethics Committee of Nanjing first hospital, and all procedures adhered to the principles outlined in the Declaration of Helsinki.

### Surgical technique

Under combined spinal-epidural or general anesthesia, patients were positioned supine with a pneumatic tourniquet. A medial approach first addressed posteromedial cortical reduction. An anterolateral approach then reduced the lateral plateau. Bone spreaders or osteotomes restored joint surface congruity and posterior tibial slope. Kirschner wires provided provisional fixation.

**Figure 1:**
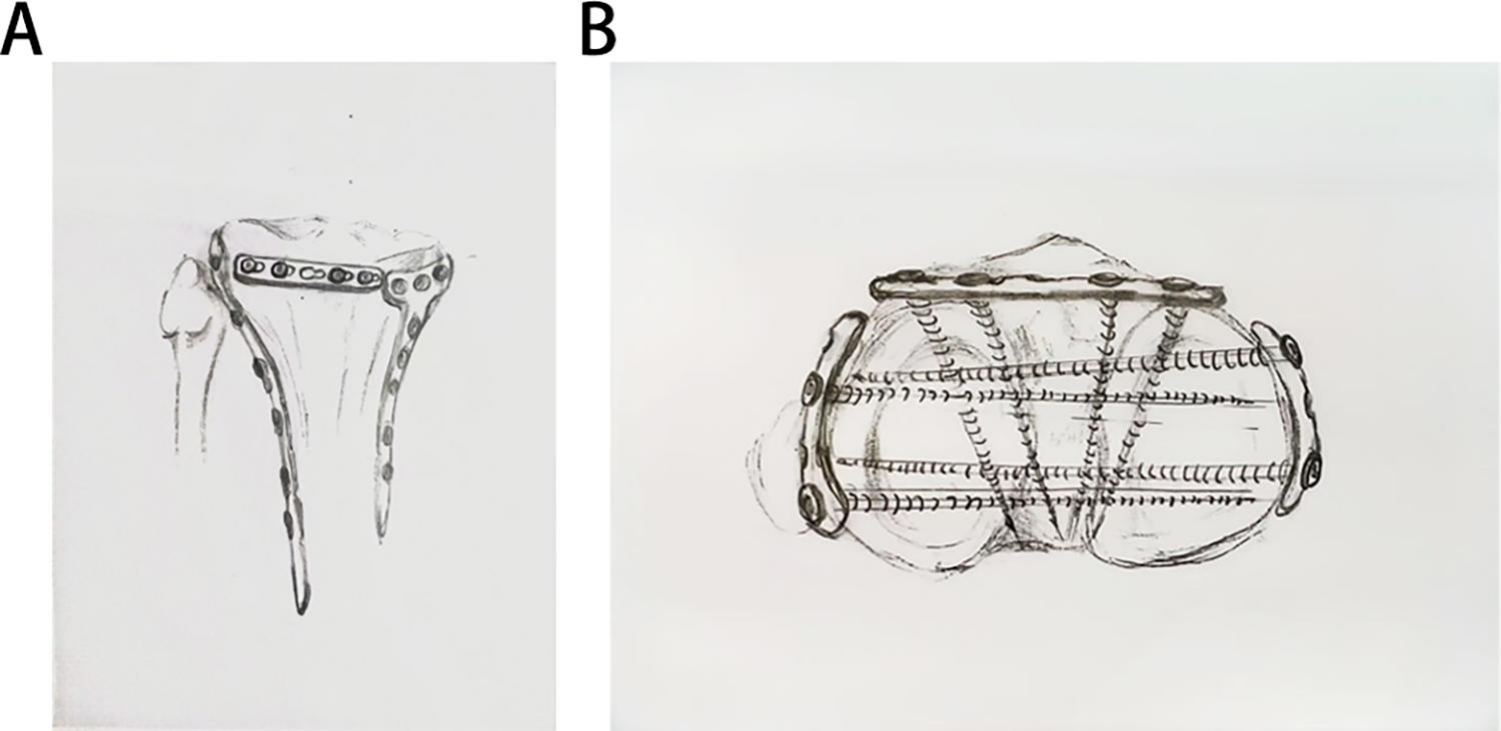
Schematic diagram of steel plate position.

**Figure 2:**
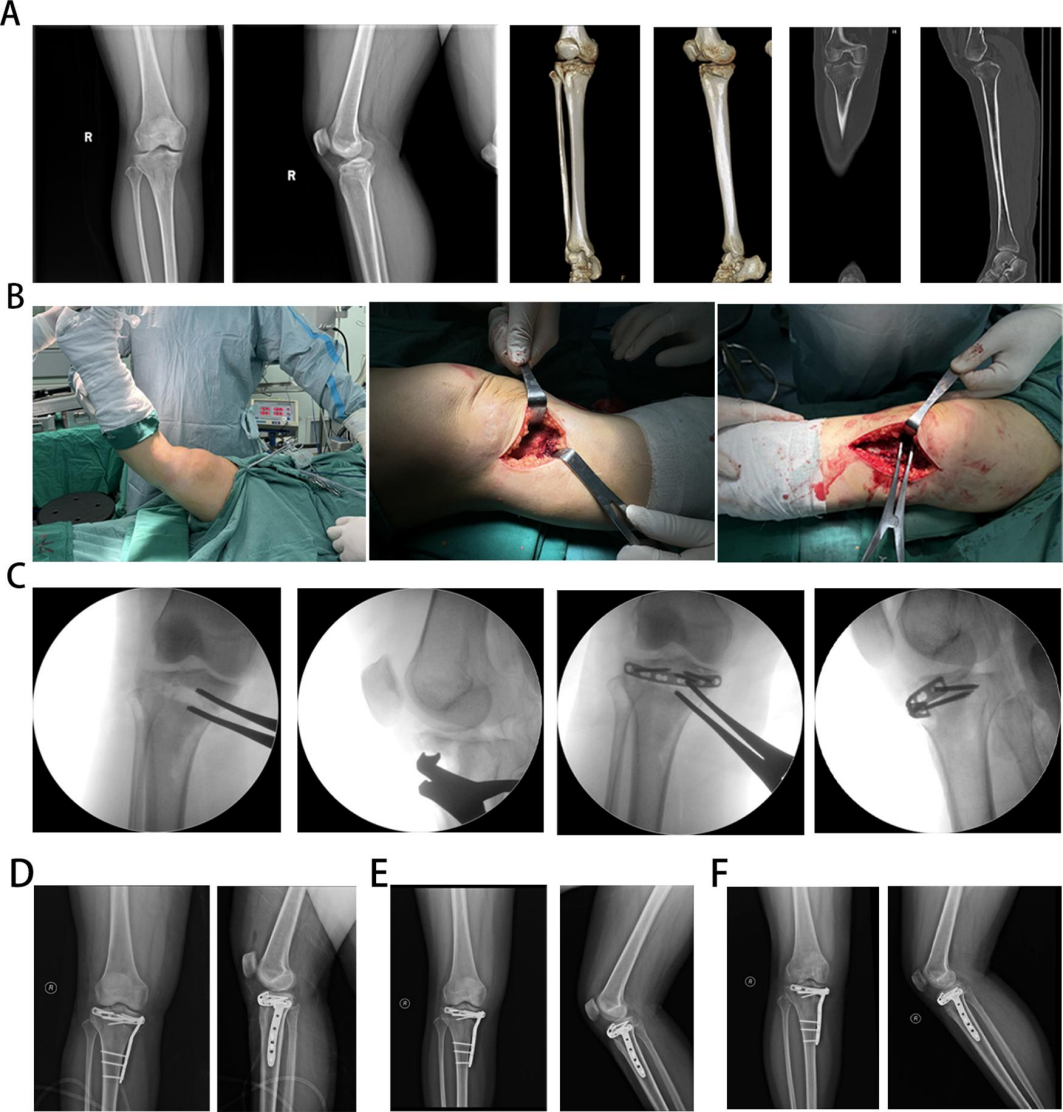
Typical surgical case images: **(A)** A 40-year-old female patient presented with bicondylar HTPF; preoperative x-ray image of patients; x-ray images of patients; preoperative 3-dimensional computed tomography imaging of patients; **(B)** intraoperative images of the surgical approach; **(C)**intraoperative C-arm fluoroscopy images. **(D)** x-ray image of patients 1 month after surgery; **(D)** x-ray image of patients 3 months after surgery.**(F)** x-ray image of patients 12 months after surgery.

A pre-contoured 1/3 tubular locking plate (Huason, Changzhou, China) was inserted beneath the patellar tendon to support the anterior “bare zone.” Medial/lateral T- or L-shaped locking plates were placed based on fracture patterns. Autologous iliac or allograft bone filled subchondral defects. Final fluoroscopy confirmed joint alignment, plate positioning, and screw lengths (Figures 1, 2).

### Postoperative management

Non-weight-bearing was maintained for 4–6 weeks, followed by progressive weight-bearing. Radiographs and CT scans assessed union. The medial tibial plateau angle (mTPA) and Posterior tibial slope (PTS) were measured on standard radiographs at both immediate post-operation and the last follow-up by two authors (Cheng Ma and Lei Zhao). Major complications were recorded, and clinical outcomes were evaluated using the hospital for special surgery (HSS) knee score at the last follow-up.

### Statistical analysis

The obtained data were statistically analyzed using SPSS 24.0 software, and measurement data were displayed as (c ± s).

## Results

All 10 patients (6 males, 4 females; mean age: 48.3 years) achieved bony union within 12–19 weeks (mean: 14.6 weeks). Mean operative time was 122 min (range: 90–180), with 220 mL average blood loss. Postoperative PTS averaged 7.8° (range: 6°–11°). HSS scores averaged 88.7 (range: 80–94), with knee flexion averaging 113° (range: 100°–130°). No infections or hardware failures occurred.

## Discussion

Hyperextension-type bicondylar tibial plateau fractures, characterized by anterior knee instability, knee recurvatum, articular surface collapse, compression of the anterior tibial cortex, and loss of posterior tilt angle, require aggressive surgical treatment to restore knee stability and reduce complications such as traumatic arthritis (8). Zeng et al. (9) utilized an anterior midline approach for hyperextension-type tibial plateau fractures, which allows direct visualization of the anterior articular surface and intercondylar fossa. However, this approach requires extensive subcutaneous dissection to expose both condyles and may lead to skin/soft tissue necrosis or infection when combined with dual or triple plate fixation. Additionally, plate placement is restricted by the patellar tendon and tibial tubercle. Although combined medial and lateral approaches are commonly used for these fractures (10), the anterior “bare area” of the tibial plateau may remain unfixed when using only proximal tibial locking plates, potentially increasing the risk of secondary reduction loss. Sun Z proposed a novel method using rim plates to support the anterior “bare area”, achieving satisfactory outcomes in a case series (7). Rim plate application in tibial plateau fractures is not uncommon due to their sufficient supporting strength and minimal space occupation. Our institution adopted a similar infrapatellar rim plating technique modified from Sun Z's method, with particular emphasis on screw positioning in medial and lateral plates to create a fence-like subchondral support structure. Evidence from biomechanical research indicates that a potential ability of the jail technique is the prevention of screw cut-outs through the cancellous bone (11). Meantime, This configuration enhances horizontal containment and provides transverse convergence fixation for comminuted articular edge fragments. The anterior horizontal rim plate reduces the need for separate anteromedial and anterolateral T-shaped plates.

Rim plates have demonstrated significant utility in tibial plateau fracture management. Pires et al. (12) reported 9 cases of posterolateral fractures treated via fibular approach with 2.7 mm horizontal rafting plates for articular support. Bermúdez et al. (13) described two high-energy bicondylar fractures with severe posterolateral comminution managed through an extended anterolateral approach using contoured 3.5 mm reconstruction plates as horizontal band plates. Cho et al. (14, 15) employed modified anterolateral approaches with rim plates for posterolateral fractures, while Zhu et al. (16) introduced a modified Frosch approach using “barrel hoop plates” as rim plate variants. Kumar (17) reported posterior medial condyle fracture fixation with rim plates, and Giordano et al. (18) developed a “ring” plate technique for posterior bicondylar shear fractures. Arthroscopy-assisted minimally invasive rim plate fixation has also been documented (19).

In hyperextension-type bicondylar fractures, dual medial and lateral approaches allow visualization of anterior platform collapse. Intraoperative use of dual distractors for reduction, combined with pre-contoured rim plates, can replace conventional anteromedial and anterolateral T-plates, reducing hardware quantity and soft tissue irritation. Strategic screw orientation creates an integrated subchondral support system with main plates, enhancing fixation stability.

Potential concerns include patellar tendon irritation from anterior plating. However, the infrapatellar fat pad may serve as a buffer, and similar to Sun Z's findings, no anterior knee pain was reported in our series. Another consideration is the biomechanical adequacy of rim plates for anterior comminution. Our technique addresses this by reinforcing fixation through engagement with relatively intact posterior and peripheral fragments, thereby enhancing overall stability.

This study has inherent limitations due to its descriptive design and the absence of a control group. Consequently, the analysis was restricted to descriptive statistics, precluding the use of comparative inferential tests. Future comparative studies are warranted to validate the findings and explore the potential advantages of this technique. Additionally, the follow-up period was insufficient to assess long-term complications, such as post-traumatic arthritis.

## Conclusion

Combined medial-lateral approaches with horizontal belt plate fixation effectively address hyperextension-type bicondylar tibial plateau fractures. Key principles include restoring limb alignment, correcting posterior slope, and anterior bone grafting. These initial findings suggest that the treatment strategy is effective and well-tolerated. Larger comparative studies are needed to validate long-term efficacy.

## Data Availability

The raw data supporting the conclusions of this article will be made available by the authors, without undue reservation.
